# IKK2 Inhibition Attenuates Laser-Induced Choroidal Neovascularization

**DOI:** 10.1371/journal.pone.0087530

**Published:** 2014-01-28

**Authors:** Huayi Lu, Qingxian Lu, Subhash Gaddipati, Ramesh Babu Kasetti, Wei Wang, Manolis Pasparakis, Henry J. Kaplan, Qiutang Li

**Affiliations:** 1 Departments of Ophthalmology and Visual Sciences, University of Louisville School of Medicine, Louisville, Kentucky, United States of America; 2 James Graham Brown Cancer Center, University of Louisville School of Medicine, Louisville, Kentucky, United States of America; 3 Second Hospital of Jilin University, Changchun, Jilin Province, P.R. China; 4 Institute for Genetics, University of Cologne, Cologne, Germany; Children's Hospital Boston, United States of America

## Abstract

Choroidal neovascularization (CNV) is aberrant angiogenesis associated with exudative age-related macular degeneration (AMD), a leading cause of blindness in the elderly. Inflammation has been suggested as a risk factor for AMD. The IKK2/NF-κB pathway plays a key role in the inflammatory response through regulation of the transcription of cytokines, chemokines, growth factors and angiogenic factors. We investigated the functional role of IKK2 in development of the laser-induced CNV using either *Ikk2* conditional knockout mice or an IKK2 inhibitor. The retinal neuronal tissue and RPE deletion of IKK2 was generated by breeding *Ikk2^−/flox^* mice with *Nestin-Cre* mice. Deletion of *Ikk2* in the retina caused no obvious defect in retinal development or function, but resulted in a significant reduction in laser-induced CNV. In addition, intravitreal or retrobulbar injection of an IKK2 specific chemical inhibitor, TPCA-1, also showed similar inhibition of CNV. Furthermore, *in vitro* inhibition of IKK2 in ARPE-19 cells significantly reduced heat shock-induced expression of NFKBIA, IL1B, CCL2, VEGFA, PDGFA, HIF1A, and MMP-2, suggesting that IKK2 may regulate multiple molecular pathways involved in laser-induced CNV. The *in vivo* laser-induced expression of VEGFA, and HIF1A in RPE and choroidal tissue was also blocked by TPCA-1 treatment. Thus, IKK2/NF-κB signaling appears responsible for production of pro-inflammatory and pro-angiogenic factors in laser-induced CNV, suggesting that this intracellular pathway may serve as an important therapeutic target for aberrant angiogenesis in exudative AMD.

## Introduction

Age-related macular degeneration (AMD) is the most common cause of irreversible blindness in humans over 55, and affects 10 to 15 million people in the USA [1]. Many factors contribute to AMD development, including age, genetic predisposition and environmental factors, such as smoking and possibly diet [2]. There are two forms of AMD, i.e., dry (non-exudative) and wet (exudative). Dry AMD is most common accounting for 90% of the disease and marked by a slow progressive degeneration of both retinal pigment epithelium (RPE) and overlying photoreceptors within the macula. In contrast, wet AMD is characterized by aberrant angiogenesis within the subretinal space, referred to as choroidal neovascularization (CNV) or within the retina, referred to as retinal angiomatous proliferation (RAP), and usually causes severe and rapid vision loss [2]. FDA-approved drugs for the treatment of wet AMD include Lucentis (Ranibizumab), Eylea (Aflibercept), and Macugen (Pegaptanib), all of which target VEGF to slow the growth of abnormal blood vessels and reverse the increased vascular permeability associated with new vessel formation [3]–[5]. These drugs, as well as Avastin (Bevacizumab), have been shown to be effective in the treatment of many patients with wet AMD but they require frequent intravitreal injections and resistance to anti-VEGF monotherapy in wet AMD is also apparent. Therefore, development of new therapeutic approaches or combination therapies is needed.

The cause of CNV is still under intense investigation and inflammation appears to be an important component. Drusen, a clinical feature associated with AMD and choroidal neovascularization, contains C-reactive protein, complement factors (C3a and C5a), and matrix metalloproteases [6]. Macrophages, lymphocytes and other inflammatory cell types are found in subfoveal CNV membranes [7]. Genetic polymorphism in complement factor H, a complement-inactivating factor, as well as other complement genes has a strong association with AMD [8]–[10]. These observations suggest that inhibition of inflammation may be a logical approach to prevent or delay the onset of AMD.

Mounting evidence indicates that NF-κB signaling plays a key role in controlling innate and adaptive immunity [11]. The NF-κB family consists of 5 structurally related proteins including p100, p105, relA, relB and c-Rel. In resting cells, NF-κB dimers are retained in the cytoplasm through association with inhibitory IκB proteins, such as IκBα. IκB stability is controlled by the IKK complex. There are two IκB kinases in the complex, namely, IKK1 and IKK2, with IKK2 being the major control for NF-κB activation; lack of this kinase blocks NF-κB activity and, in turn, the pro-inflammatory response [12], [13]. Activation of IKK2 causes IκB phosphorylation that leads to ubiquitin-dependent protein degradation, and thus, releases IκB-bounded NF-κB from the cytoplasm. Nuclear localization of NF-κB activates the transcription of many cytokine, chemokine, and growth factors genes [13].

**Figure 1:**
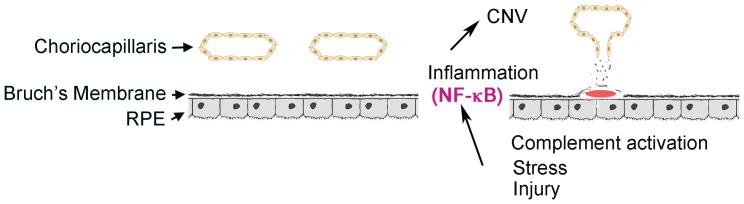
Schematic diagram shows targeting strategy by inhibiting IKK2/NF-κB pathway to prevent choroidal neovascularization (CNV) in AMD. Chronic inflammation and oxidative stress caused by pathologic changes, tissue damage or local components activation in subretinal space induce expression and secretion of cytokines, growth factors and extracellular matrix molecules by RPE, which in turn initiate CNV. NF-κB is a key transcription factor necessary for inflammatory and stress responses. Inhibition of NF-κB activation by IKK2 inhibitor is postulated to prevent chronic inflammation- and oxidative stress-induced CNV formation.

We hypothesize that oxidative stress and hypoxia during aging induce sustained IKK-NF-κB activation and chronic inflammation in the RPE and choroid, leading to up-regulation of angiogenic factors and aberrant neovascularization (Fig.1). To assess the functional role of IKK2 in aberrant angiogenesis, we investigated the effect of *Ikk2* gene deletion or chemical inhibition of IKK2 activity on development of laser-induced CNV, a murine model of wound healing accompanied by oxidative stress and inflammation [14], [15]. Since total *Ikk2* knockout mice die *in utero* between E12.5 and E14.5 due to liver apoptosis, we generated conditional knockout mice by breeding the *Ikk2^−/flox^* mice with *Nestin-Cre* mice to create deletion of *Ikk2* in the RPE and retinal neurons. Deletion of functional IKK2 in the retina did not affect normal retinal development or function, but significantly reduced laser-induced CNV. Similarly, intravitreal or retrobulbar injection of an IKK2 specific chemical inhibitor, 2-[(aminocarbonyl)amino]-5-(4-fluorophenyl)-3-thiophenecarboxamide (TPCA-1), caused a similar reduction in CNV. Both *in vitro* and *in vivo* experiments showed that TPCA-1 treatment inhibited injury-induced expression of multiple genes, suggesting that the anti-angiogenic effect of IKK2 inhibition involved multiple molecular pathways. Our data showed that NF-κB signaling, through regulation of pro-inflammatory and pro-angiogenic factors, played an important role in the development of laser-induced CNV. We propose that the IKK2/NF-κB pathway may serve as a novel therapeutic target in the prevention or treatment of wet AMD.

## Materials and Methods

### Ethics statement

All animal experiments (Protocol: 12010) were approved by Institutional Animal Care and Use Committee, the Animal Care Committee at the University of Louisville, and were conducted in accordance with the guidelines of Association for Research in Vision and Ophthalmology on the use of animals in research.

### Animals

The *Ikk2^+/−^* mice and *Ikk2^flox/flox^* mice were generated and described previously [12], [16]_ENREF_32_ENREF_27. The *Nestin-Cre^+^* transgenic mice (stock # 003771) [17], the *mT/mG* (membrane-Tomato/membrane-Green) homozygous transgenic mice (stock # 007676) [18], and the wild type control C57BL6J mice (stock # 000664) were purchased from the Jackson Laboratory. Compound lines of the *Ikk2^−/flox^/Nestine-Cre^+/−^* and control *Ikk2^+/flox^/Nestine-Cre^+/−^* mice, were generated by crossing *Ikk2^+/−^/Nestin-Cre^+/+^* with *Ikk2^flox/flox^* mouse lines. The *Nestin-Cre^+/−^/mT/mG^+/−^* and *control mT/mG^+/−^* mice were generated through the cross-breeding of the *Nestin-Cre^+/−^* mice to the *mT/mG^+/+^* homozygous transgenic mice. All animals were maintained and used in accordance with the guidelines of Association for Research in Vision and Ophthalmology on the use of animals in research and according to the guidelines of the Animal Care Committee at the University of Louisville.

### Laser treatment

The laser-induced neovascularization was performed on mice at ages between 6–10 weeks. The pupils were dilated with a single drop mixture of 0.06% tropicamide and 0.3% phenylephrine hydrochloride. Two minutes later, the mice were anaesthetized with intraperitoneal injection of Ketamine-Xylazine solution (Ketamine at 15 mg/mL saline and Xylazine at 6 mg/mL saline) at 0.05 mL per 10 g body weight. Diode laser burns applied with a Novus Spectra ophthalmic laser (Lumenis, Inc., Santa Clara, CA) mounted on a slit lamp (Model SL-M; Zeiss, Inc., Tokyo, Japan) generated four lesions symmetrically surrounding the optic nerve of each eye. A coverslip coated with 2.5% hypromellose ophthalmic demulcent solution was held on the mouse cornea, which served to subtract the optics of the cornea and lens for optimal view of the retina and spots of the laser lesions [19]. The laser variables included a 50 μm spot size, 0.05 second-duration, and power at 250 mW. The power used was assessed by the ability to produce a blister indicating rupture of the Bruch's membrane. Laser spots were evaluated by fluorescein angiography and dextran green or isolectin IB4 staining for the presence of CNV at days 7 and 10 after laser treatment.

### Retrobulbar injection

Under deep anesthesia, mouse was placed on its side, and a beveled G33 needle was inserted at an approximately 45° angle into the eye, from lateral to the medial canthus, through the conjunctiva membrane. The needle was positioned behind the globe of the eye in the retrobulbar sinus after piercing through the conjunctiva. The mice received unilateral retrobulbar injection of 20 μL of 10 mM IKK2 inhibitor TPCA1 in PBS containing 20% dimethyl sulfoxide (DMSO). Retrobulbar injection of 20 μL vehicle buffer (PBS/20% DMSO) served as control.

### Intravitreal injection

Right after laser injury, the mouse eye was decompressed by inserting a 30G needle through the conjunctiva and sclera at 1 mm behind the limbus. Microinjector UMP3 equipped with Nanofil syringe 100 μL and 33G blunt needle (Word precision instruments, Sarasota, FL) was used for injection. The needle was positioned firmly when the operator felt a slightly resistance and then 2 μL of solution was slowly injected in 10 seconds. The topical Ocuflox drops were applied after procedure to prevent infection.

### Optokinetic reflex (OKR) measurements

Visual function was assessed using a non-invasive OptoMotry© optokinetic testing system (CerebralMechanics, Lethbridge, AB, Canada), following the procedures described and validated previously [20], [21]. Briefly, mice standing unrestrained on the central platform tracked a rotating grating with reflexive head movement behavior. Testing was initiated by projecting a grating of low spatial frequency [0.042 cycles/degree (c/d)], rotating at 12 degrees/second at maximum 100% contrast, i.e. the bars of the gratings are maximally black and the background is maximally white. Spatial frequency of the grating was increased until the animal no longer responded. The threshold of maximum spatial frequency that the mouse could track is a correlate of visual acuity. OKR could detect visual function for each eye separately. OKR response to the rotation of the gradient in a clockwise direction is dependent upon the left eye, whereas rotation in a counterclockwise direction is dependent upon the right eye.

### Fluorescein angiography

*In vivo* fundus fluorescein angiography of CNV was conducted using a retinal camera (model TRC 50X; Topcon America Corp., Paramus, NJ) with Canon 5D digital imaging hardware. The pupil dilation and anesthesia were performed as describe above. Fluorescein fundus images were taken at 3–6 min after intraperitoneal injection of 0.3–0.5 mL of 100 mg/mL Fluorescein (10% w/v). Lesion sizes were graded on the spatial comparison between the fluorescein leakage area and the optic nerve disc area [22], 0: no leakage; 1: the area of fluorescein leakage was equal to disc area, 2: the area of fluorescein leakage was approximately double to the disc area.

### Fluorescein-labeled dextran staining and isolectin IB4 staining to evaluate CNV area

Under deep anesthesia, the mouse right atrium was cut and the blood immediately was washed for 3 min with 0.5 mL/min of PBS through the left ventricle with assistance of a perfusion pump, and followed by syringe injection of 1.5 mL PBS containing 50 mg/mL fluorescein-labeled dextran (FITC-dextran, 2 million average molecular weight, Sigma-Aldrich, St. Louis, MO), and then perfusion-fixed with 4% paraformaldehyde (PFA) for 6 min. The eyes were removed and immerse-fixed in 4% PFA at 4°C for overnight. After rinsed in PBS, cornea, lens, and the neurosensory retina were carefully removed from the eyecup. Five radial cuts were made from the edge of the eyecup to the equator; the RPE/choroid/sclera complex was flat-mounted in 50% glycerol containing PBS with the sclera facing down on a glass slide. A laser spot with green vessels was scored as CNV-positive, and a laser spot lacking green vessels was scored CNV-negative. The flat-mounted retina was imaged at 5× magnification using a Zeiss Axio Imager M2 system equipped with Apotome, and attached to both AxioCam and AxioVision cameras. The free hand selection tool was used to delineate the boundaries of CNV complexes and the total area of each lesion across all sections was measured and expressed in square micrometers (μm^2^) to determine maximum CNV complex area. The sum of CNV area in each eye was compared among different animals and experimental conditions.

For isolectin IB4 staining, laser injured eyes were enucleated and fixed at 4°C with ice-cold 4% paraformaldehyde in PBS for overnight. The anterior segment, lens, and retina were removed, and the remaining eye cups with attached RPE layer were washed with PBS and incubated for overnight at room temperature with1∶100 dilution of 1 mg/mL solution of AlexaFluor 594 conjugated isolectin IB4 (Invitrogen, Oregon, USA) [23]. After incubation, the eye cups were washed with PBS, the sclera-choroid/RPE complexes with radial cut were flat mounted for fluorescence microscopy right after the staining. The area of fluorescence was quantified as described above.

### Histological studies

The eyes were dissected from euthanized mice and fixed immediately in 4% PFA for overnight at 4°C, and then subjected to either paraffin embedding or OCT embedding for Cryostat sectioning. Seven μm of paraffin-embedded sections was cut and de-paraffinized prior to staining with hematoxylin and eosin (H&E). The mean thickness of the CNV membrane was obtained by examining the serial sections of whole eyes and using the maximum thickness measurements of each of the four lesion sites per eye. Frozen tissue sections from the *Nestin-Cre^+/−^/mT/mG^+/−^* and control *mT/mG^+/−^* mice were examined for the switch from red fluorescence to green fluorescence using Zeiss Axio Imager M2 system equipped with ApoTome, and attached with AxioCam and AxioVision cameras. The cell layers of the inner nuclear layer and outer nuclear layer were counted by random sampling 10 sagittal sections from each eye.

### Western blot analysis

Skin, brain and retina tissues from *Ikk2^−/fl^/Nestine-Cre^+/−^* and WT (*Ikk2^+/fl^*) control mice were collected and homogenized in cold immunoprecipitation assay buffer (20 mM Tris-HCl, pH 7.4, 100 mM NaCl, and 0.2% each of deoxycholate, Triton X-100, and Nonidet P-40) containing 1× complete protease-inhibitor mixture (Roche Diagnostics, Indianapolis, IN). Equal amounts of whole-cell lysates were separated on 10% sodium dodecyl sulfate polyacrylamide gel (SDS-PAGE) and transferred to a nitrocellulose membrane. For immunoblotting, the mouse anti-IKK2 antibody (Biosource AH00362, 1∶100 dilution), and mouse anti-β-actin (1∶1000, cat #A2228, Sigma, St. Louis, MO) were used as primary antibodies. The horseradish peroxidase-conjugated goat-anti-mouse secondary antibody and an enhanced chemiluminescence (ECL) system (Amersham Pharmacia, Piscataway, NJ) were used to visualize the signals. For immunobloting of IκBα and α-tubulin in the ARPE-19 cells pre-treated with TPCA-1 and TNFα, rabbit anti-IκBα antibody (1∶250 dilution, Santa Cruz Biotechnology, Inc, sc-371) and mouse anti-α-tubulin antibody (1∶500 dilution, Sigma, T9026) were used. For thermal injured ARPE-19 cells, rabbit-anti-NF-κB p65 antibody (1∶200 dilution, Santa Cruz Biotechnology, Inc., sc-372) and rabbit-anti-NF-κB p65 (Ser536) antibody (1∶500 dilution, Cell Signaling Technology, Inc., Cat #3033) were used.

### RNA isolation and Quantitative (q)-PCR

The total RNA from the spleens, brains and retinas of *Ikk2^−/flox^/Nestine-Cre^+/−^* and WT control mice were extracted using TRIzol reagent (Invitrogen). The total RNA from the RPE/Choroid/Sclera tissues of laser injury eyes was extracted using TRIzol reagent (Invitrogen) after removing the anterior segment, vitreous, and retina. The A260/A280 ratio of all RNA samples was >2.0 as measured by Nanodrop. Double-stranded cDNA was reverse-transcribed using random primers and SuperScript VILO cDNA synthesis kit (Invitrogen). Real time qPCR by a SYBR green-based PCR method was performed on a MX3005p system (Agilent Technologies, Inc., Santa Clara, CA), with a program of a 10-minute initial hot-start activation of Taq polymerase at 95°C, followed by 40 cycles of amplification (95°C for 10 seconds, 56°C for 5 seconds, and 72°C for 10 seconds). The comparative CT (threshold cycle) method normalized to β-actin was used to analyze relative changes in gene expression. The primers for human NFKBIA (IκBα) (ID 10092619a1), IL1B (Il1β) (ID 27894305b1), CCL2 (ID 4506841a1), VEGFA (ID 30172564a1), PDGFA (ID 15208658a1), HIF1A (ID 4504385a1), MMP2 (11342666a2), mouse Hif1a (ID 7363433a1), and Hmox1 (6754212a1) were designed based on the online PrimerBank database (Harvard Medical School, Boston, MA). The qPCR primer sequences for human ACTB (beta-actin), mouse Actb(beta-actin) and mouse Ikbkb (Ikk2) have been published elsewhere [24], [25]. The detailed primer information is listed in Table 1.

**Table 1:** List of primers used for qPCR.

Gene	Primer sequence
Mouse *Ikbkb*	F-5′-GGAGTACTGCCAAGGAGGAGAT-3′R-5′-ACAGGCTGCCAGTTAGGGAGGAAG-3′
Mouse *Actb*	F-5′-CCAGTTGGTAACAATGCCATGT-3′R-5′-TGT ATG CTA TAC GAA GTT AT-3′
human *ACTB*	F-5′-AACTCCATCATGAAGTGTGACG-3′R-5′-GATCCACATCTGCTGGAAGG-3′
human *NFKBIA*	F-5′-CTCCGAGACTTTCGAGGAAATAC-3′R-5′-GCCATTGTAGTTGGTAGCCTTCA-3′
human *IL1B*	F-5′-ATGATGGCTTATTACAGTGGCAA-3′R-5′-GTCGGAGATTCGTAGCTGGA-3′
Human *CCL2*	F-5′-CAGCCAGATGCAATCAATGCC-3′R-5′-TGGAATCCTGAACCCACTTCT-3′
Human *VEGFA*	F-5′-CAACATCACCATGCAGATTATGC-3′R-5′-GCTTTCGTTTTTGCCCCTTTC-3′
Human *PDGFA*	F-5′-CCAGCGACTCCTGGAGATAGA-3′R-5′-CTTCTCGGGCACATGCTTAGT-3′
Human *HIF1A*	F-5′-GGCGCGAACGACAAGAAAAAG-3′R-5′-CCTTATCAAGATGCGAACTCACA-3′
Human *MMP2*	F-5′-GCCCCAGACAGGTGATCTTG-3′R-5′-GCTTGCGAGGGAAGAAGTTGT-3′
mouse *Vegfa*	F-5′-ACAGCACAGCAGATGTGAATGCAG-3′R-5′-TTACACGTCTGCGGATCTTGGACA-3′
mouse *Hif1a*	F-5′-ACCTTCATCGGAAACTCCAAAG-3′R-5′-CTGTTAGGCTGGGAAAAGTTAGG-3′
mouse *Hmox1*	F-5′-AAGCCGAGAATGCTGAGTTCA-3′R-5′-GCCGTGTAGATATGGTACAAGGA-3′

### TNFα induced IκBα degradation and TPCA-1 treatment on ARPE-19 cells

ARPE-19 cell, a human RPE cell line, was cultured according to supplier's instruction (ATCC, catalog CRL-2302, Manassas, VA). When reaching 90% confluence in 3.5 cm plates, the cells were pretreated with TPCA-1 (Tocris Bioscience, Ellisville, Missouri) at 0, 2, 20 and 200 μM for 30 minutes, and then added with TNFα at 10 ng/mL for additional 15 or 30 minutes. Control samples are treated with TPCA-1 only. Protein lysates were subjected to Western blotting for detection of IκBα degradation.

### Thermal injury experiments

The confluent ARPE-19 cells cultured on 3.5 cm plates were pretreated with or without 5 μM TPCA1 (Tocris Bioscience, Ellisville, Missouri) for 30 minutes prior to thermal injury. The thermal injury experiments were carried out by replacing the medium in cell plates with 10 mL HEPES-buffered saline pre-warmed at 55°C for 5 seconds before changed back to normal culture medium at 37°C in the presence or absence of 5 μM TPCA1. Cells were harvested at 10, 30, 60, 120 minutes after heat shock for protein preparation and the total RNA for qPCR was isolated using TRIzol reagents at 4 hours after heat shock.

### Luciferase assay

293/NF-κB-Luc cells (Cat # RC0014, Affymetrix, Inc., Santa Clara, CA) were plated in poly-lysine coated 96-well plates. The cells at 90% confluence were pretreated with 0, 1, 2.5, 5, 10 μM TPCA1 for 30 minutes, then stimulated with 10 ng/mL TNFα (PeproTech Inc., Rocky Hill, NJ) in the presence of indicated amount of TPCA-1 (Tocris Bioscience, Ellisville, Missouri) for 5 hours, and followed by protein lysate collection and luciferase assay. TPCA-1 and TNFα untreated sample used as controls. Luciferase assays were performed using Luciferase assay kit (E1910, Promega, Madison, WI) following the manufacturer's protocol.

### MTT cell viability assay

The HEK293T and ARPE-19 cells were cultured in poly-lysine coated and uncoated 96-well plates, respectively. The cells at 60–70% confluence were treated in the following 3 conditions, i.e., 50 μM TPCA1 alone, 10 ng/mL TNFα alone or in combination of both TPCA-1 and TNFα after a 30 minutes pretreatment of 50 μM TPCA1. Cell without any drug treatment served as controls. The cell viability after 12 hours of drug treatment was measured by a MTT assay. Briefly, 0.2 mL of 3-[4,5-dimethylthiazol-2-yl]-2,5-diphenyltetrazolium bromide (MTT) at 5 mg/mL in PBS was added to each well and the cells were incubated at 37°C for 4 hours. After the medium was removed, the purple formazan precipitate was solubilized in 200 μL DMSO. Absorbance was measured at 560 nm with background subtraction at 670 nm. Measurements were made in triplicate and the error bars represent the standard deviation. The Student's two-tailed *t*-test was used to determine statistical significance.

### Flow cytometry

Flow cytometry was performed as described previously [26]. Briefly, the single cell suspensions were prepared from thymus, spleen, and bone marrow of the mice at 7 days after receiving a single retrobulbar injection of 20 μL of 10 mM (56 μg) TPCA-1 in PBS or control vehicle, and passed through 70-μm nylon mesh (Fisher Scientific). After lysis of RBCs using ACK buffer (0.83% ammonium chloride, 0.1% potassium bicarbonate, 0.037% EDTA [pH 7.2]), Fc receptors were blocked with anti-CD16/32 antibody (Ab) before staining. To stain cell surface antigens, 10^6^ cells in 50 μL of staining buffer (1×PBS containing 3% BSA and 0.1% sodium azide) were incubated at 4°C for 30 minutes with fluorochrome-conjugated mAbs, and analyzed on a four-color BD FACSCalibur (BD Biosciences), the data was analyzed with CellQuestPro 5.1.1 software (BD Biosciences). Antibodies used for staining were as follows: rat anti-mouse CD16/32 blocking Ab (clone 2.4G2) was obtained from BD Biosciences (San Diego, CA); and rat anti-mouse TCR-β-FITC (H57-597), CD8a-PE (53-6.7), CD4-APC (GK1.5), B220-PE (RA3-6B2), IgD-APC (11-26), IgM-FITC (eB121-15F9), CD11b-APC (M1/70), Ly6C-PE (HK1.4), Ly6G-FITC (RB6-8C5), 7-amino-actinomycin D (7-AAD), and PE-conjugated rat IgG2a or IgG2b isotype Ab controls were purchased from eBiosciences (San Diego, CA).

### Statistical analysis

The quantitative data were presented as means ± standard deviation (SD). CNV spot number, area, and choroidal neovascular membranes between the mutant and WT groups or drug treated and vehicle-treated controls were compared by performing a two-tail Student's t-test on raw data. P-values of less than 0.05 were considered to be significant.

## Results

### IKK2 is not essential for normal retinal development and function

**Figure 2:**
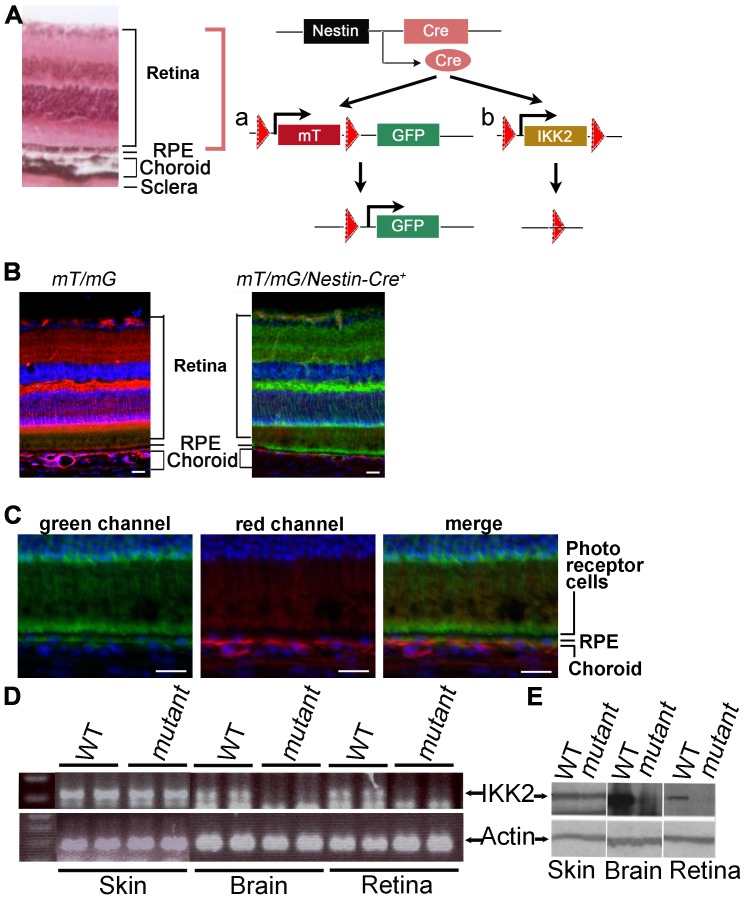
IKK2 is deleted in the RPE and retinal neurons in *Ikk2^−/flox^/Nestin-Cre^+^* mice. (A) Nestin-promoter-driven Cre mediated specific deletion of the LoxP flanked genomic sequence was tested on mT/mG/Nestin-Cre^+^ mice (a) and performed on *Ikk2^−/flox^/Nestin-Cre^+^* mice (b). The *mT/mG* (membrane-Tomato/membrane-Green) mouse strain contains a Cre-reporter transgene knocked into the Rosa26 locus. When bred with the *nestin-Cre* mice, the Cre recombinase mediated the genomic deletion of the LoxP (solid triangles)-flanked tdTomato (red) STOP cassette and in turn led to expression of the downstream membrane-targeted enhanced green fluorescent protein (GFP). A similar nestin-Cre mediated homologous recombination to obtain a neuronal and RPE deletion of *Ikk2* in retina was designed by breeding the *Ikk2^−/flox^* mouse carrying one *Ikk2* mutant allele and one *Ikk2*-flox allele with the mouse line expressing *Cre* recombinase under control of *Nestin* promoter (*Nestin-Cre*). The hematoxylin- and eosin-stained wild type mouse eye section on left show anatomy of retina, choroid, and sclera. (B, C) The RPE and neuronal retinal cell deletion of the LoxP-flanked tdTomato STOP cassette mediated by *Nestin-Cre* was confirmed by the red-to-green fluorescent color conversion on the retinas of the *mT/mG/Nestin-Cre^+^* cre reporter mice, whereas the adjacent choroid tissue did not show such red-to-green change, as shown at lower (B) and higher (C) magnification images. Red cells represent cre-negative cells, while green cells are cre-positive cells. (D) Reverse transcription PCR showed that *Ikk2* mRNA was eliminated in both retina and brain, but not in the control tissue, i.e., the skin of the *Ikk2^−/flox^/Nestin-Cre^+^* mice. (E) Western clot analysis showed that the IKK2 protein was absent in the retina and brain, but not in the skin of the *Ikk2^−/flox^/Nestin-Cre^+^* mice. Size bar = 20 μm in B and C.

To examine the functional role of IKK2 in development of laser-induced CNV, we generated conditional *Ikk2* mutant mice by breeding *Ikk2^−/flox^* mice carrying one *Ikk2* mutant allele and the *Ikk2-flox* allele [27] with the mouse line expressing *Cre* recombinase under control of the *Nestin* promoter (Fig. 2A). To directly visualize the neuronal and RPE deletion of the loxP-flanked sequence in the eye by the *Nestin* promoter-driven *Cre* recombinase, we first bred *Nestin-Cre* mice into *mT/mG* (membrane-Tomato/membrane-Green) dual color fluorescent Cre-reporter mice. GFP expression is blocked in these mice by a loxP flanked red fluorescent protein (tdTomato) expression cassette in non-Cre cells, but allowed in cells expressing Cre after the Cre-mediated deletion of the loxP flanked tdTomato expression cassette. This double-fluorescent system allows direct visualization of Cre positive (green) and Cre negative (red) cells in the retina. Our data clearly showed that Nestin-Cre caused a selective loxP flanked genomic recombination in retinal neurons and RPE cells but not in choroidal tissue (Fig. 2B). Very few red colored cells were detected in the neurosensory retina, indicating that the Nestin-Cre mediated recombination was highly efficient (Fig. 2C). Significant reduction of *Ikk2* expression at the mRNA and protein level was detected in the retina and brain, but not skin, by RT-PCR and Western blotting after Nestin-Cre mediated deletion of the loxP-flanked *Ikk2* allele (Fig. 2D). These results indicated that *Ikk2* was efficiently knocked out in the RPE and retinal neurons in the *Ikk2^−/flox^/Nestin-Cre^+^* mice.

**Figure 3:**
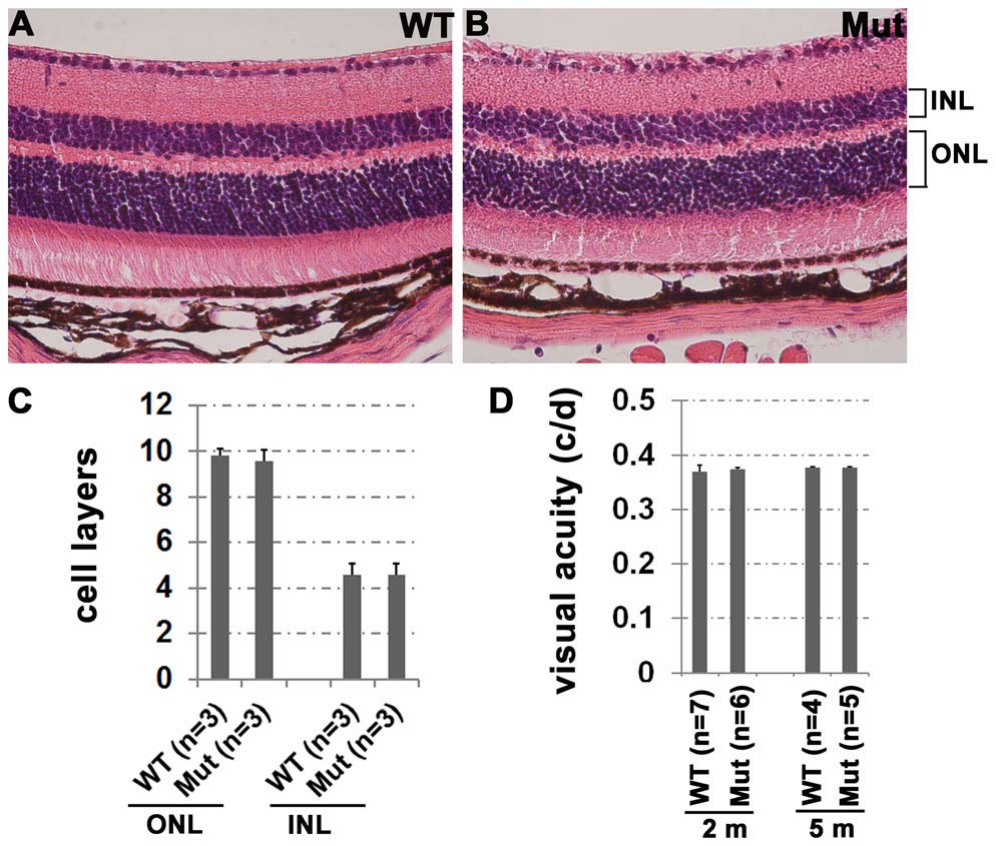
IKK2 is not required for retina development and function. (A, B) The paraffin-embedded retinal sections from the wild type control (WT) (A) and *Ikk2^−/flox^/Nestin-Cre^+^* (Mut) (B) mice at age of 2 month old were stained by hematoxylin and eosin (H&E) method. (C) The quantitative comparison of the cell layers in the outer nuclear layer (ONL) and inner nuclear layer (INL) between WT (n = 3 mice) and Mut (n = 3 mice) mice at 2 month old. (D) Visual acuity was tested on wild type (WT) control and *Ikk2^−/flox^/Nestin-Cre^+^* (Mut) mice at the age of 2 and 5 month old by OKR. Error bars indicate standard deviation.

After confirmation of the conditional knockout of the *Ikk2* gene in retinal neurons and RPE cells, we first evaluated retinal development and found no overt morphological defect in the *Ikk2^−/flox^/Nestin-Cre^+^* retina by light microscopy (Fig. 3A and 3B). Both WT and *Ikk2^−/flox^/Nestin-Cre^+^* retinas showed approximately 9–10 rows of nuclei in the outer nuclear layer (ONL) and 5 rows of nuclei in the inner nuclear layer (INL). The average ONL cell layers for WT and mutant mice were 9.8+0.32 and 9.6+0.48, respectively (Fig. 3C). The average INL cell layers for WT and mutant mice were 4.6+0.48 and 4.5+0.5, respectively. There were no significant differences in OKR spatial frequency thresholds [38] between *Ikk2^−/flox^/Nestin-Cre^+^* and WT control mice, suggesting normal visual acuity in *Ikk2^−/flox^/Nestin-Cre^+^* mice (Fig. 3D). These results indicate that IKK2 is not essential for normal retinal development or function.

### Loss of *Ikk2* inhibits laser-induced CNV

**Figure 4:**
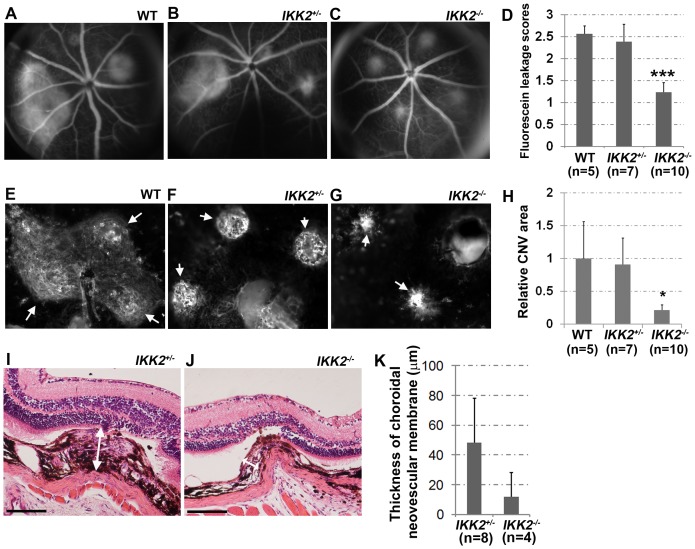
Induction of the laser-induced CNV is inhibited in the *Ikk2^−/flox^/Nestine-Cre^+^* mice. The development of CNV after laser photocoagulation was quantified by scoring the fluorescence leakage and isolectin IB4 stain areas and measuring the thickness of choroidal neovascular membrane. (A–C) Fundus photographs and fluorescein angiogram (A–C) were taken from the wild type (WT), *Ikk2^+/flox^/Nestine-Cre^+^* (*Ikk2^+/−^*), and *Ikk2^−/flox^/Nestine-Cre^+^* (*Ikk2^−/−^*) mouse eyes that had been treated with laser injury for 7 days. The development of CNV was quantified by scoring the relative average size of fluorescence leakage in each animal (D) comparing to optic disk size. N = number of mice. (E–G) The images of Alexa Fluor 568 conjugated isolectin IB4 stained CNVs were taken from the indicated mouse eyes at 7 days post-laser injury. Arrows indicate the CNV identified by isolectin IB4 staining. (H) The summary of the mean CNV area from each genotypic animal was shown as relative to that induced in the WT eyes. N = number of mice. (I and J) Representative H&E stained eye sections show the neovascular membrane networks originating from the choroicapillaries passing through the broken Bruch's membrane. The white arrows with two arrowheads show the thickness of choroidal neovascular membrane. The scale bar represents 50 μm in length. (K) Quantification of the thickness of the choroidal neovascular membrane. Error bars indicate standard deviation. Two-tail T-test: * *p*<0.05, *** *p*<0.005. n indicates the number of laser-injured spots.

To determine whether the absence of *Ikk2* influences choroidal neovascularization *in vivo*, we adopted a laser-induced CNV model [19], and evaluated the incidence of fluorescein leakage on fluorescent angiograms (FA) performed on the *Ikk2^−/flox^/Nestine-Cre^+^* (*Ikk2^−/−^*) retina on day 7 after laser treatment, compared to laser-induced CNV in control WT and *Ikk2^+/flox^/Nestine-Cre^+^* (*Ikk2^+/−^*) mice (Fig. 4A–C). Fluorescein fundus images were taken 3–6 minutes after intraperitoneal injection of 0.3 mL of 100 mg/mL Fluorescein (10% w/v). During this period, the size of fluorescein leakage spots was relatively stable and the area of each fluorescent spot was measured and expressed as arbitrary unit in relevant to the area of the optic nerve disc (Figure S1 in File S1). Newly formed microvessels with clear evidence of fluorescein leakage were observed in 63% of the laser-induced lesions in WT and *Ikk2^+/flox^/Nestine-Cre^+^* mice, compared to 35% in *Ikk2^−/flox^/Nestine-Cre^+^* mice. The average score of fluorescein leakage was reduced from 2.5 in WT mice and 2.4 in *Ikk2^+/flox^/Nestine-Cre^+^* mice to 1.2 in *Ikk2^−/flox^/Nestine-Cre^+^* mice (Fig. 4D).

CNV formation was further visualized by AlexaFluor 594-labeled isolectin IB4 staining (Fig. 4E–G). Although there was no significant difference in CNV area between WT and heterozygous *Ikk2^+/flox^/Nestine-Cre^+^* mice (Fig. 4H), complete knockout of both *Ikk2* alleles in the retina of *Ikk2^−/flox^*/*Nestin-Cre^+^* mice resulted in a 5-fold reduction in the size of CNV lesions compared to control mice (Fig. 4H). Furthermore, the thickness of the neovascular membrane was quantified by examining serial sections and using the maximum thickness measurements as indicated as in Fig.4I–J; an 80% reduction was observed in *Ikk2^−/flox^/Nestin-Cre^+^* mice compared to *Ikk2^+/flox^/Nestine-Cre^+^* mice (Fig. 4I–K). These data showed that there was a significant reduction in the size and thickness of the CNV complex in *Ikk2^−/flox^/Nestin-Cre^+^* mice, suggesting that IKK2 plays a critical role in aberrant angiogenesis.

### IKK2 chemical inhibitors reduce laser-induced CNV

**Figure 5:**
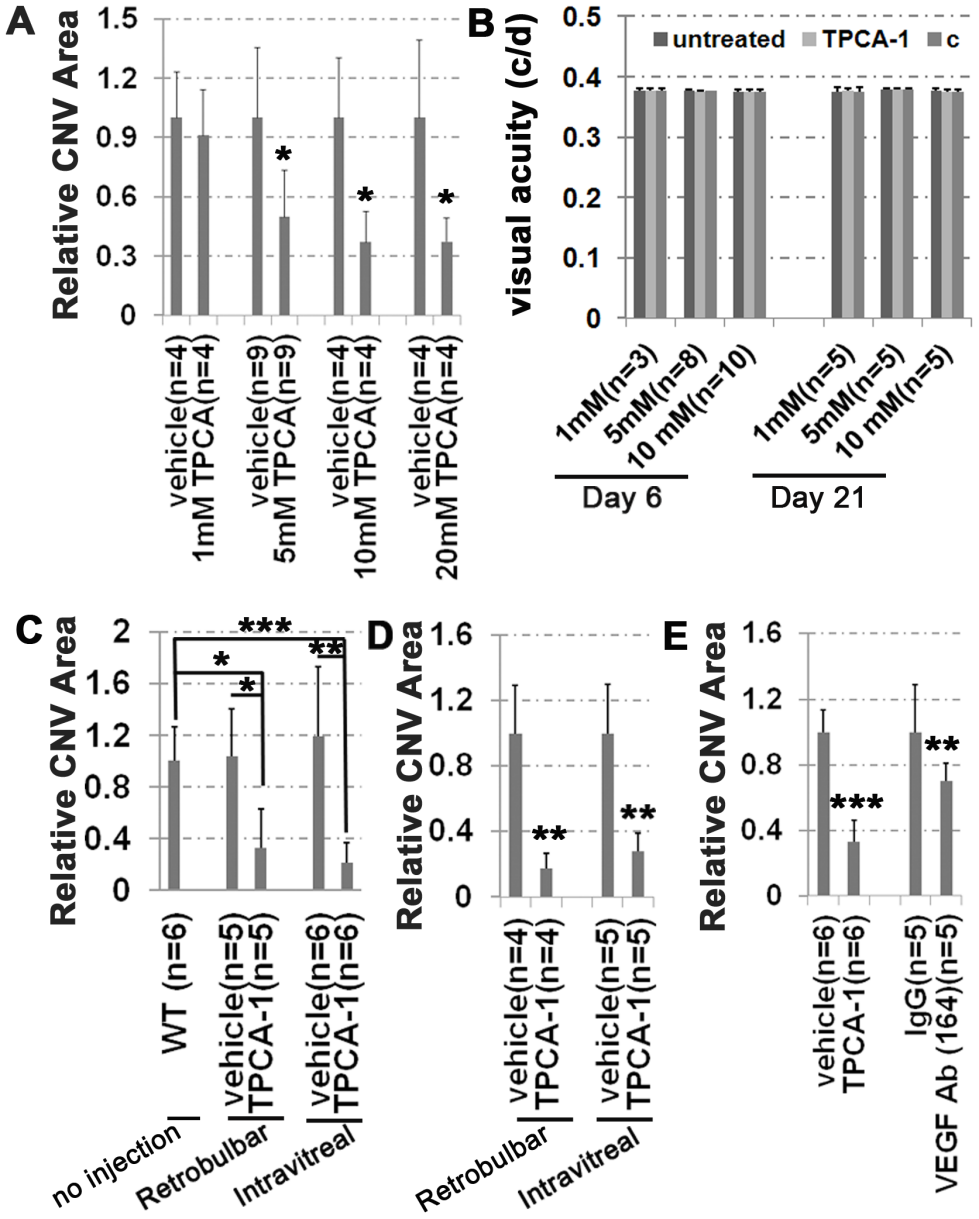
IKK2 chemical inhibitor TPCA-1 significantly reduces the laser-induced CNV formation. (A) The size measurements on the laser induced vascular sprouting in the eyes injected with 20 μL TPCA-1 or control vehicle (PBS/20% DMSO). 20 μL of IKK2 chemical inhibitor TPCA-1 at indicated concentration was retrobulbarly injected immediately after the laser injury. CNVs were visualized by FITC-dextran staining 7 days after laser injury and quantified as area size relative to CNV area in the control mice injected with vehicle solution. (B) Visual function was measured by OKR tests performed on the laser-injured and TPCA-1 treated mice on day-6 and -21 post laser injury. The mice received a retrobulbar injection of 20 μL TPCA-1 at the concentrations indicated under the X-axis of the graph in one eye right after laser injury. “Untreated” represents the results from the eyes without any injection. “TPCA-1” represents the visual acuity from the eyes retrobulbarly injected with 20 μL TPCA-1. “c” represents the combined visual acuity test results from both TPCA-1 treated and untreated eyes. (C) The size of CNV area developed in the laser-induced and TPCA-1 treated eyes was normalized to the size of CNV area in the laser-induced WT eyes that had not been given any injection after laser injury. The TPCA-1 treatment was given once to the mice via either retrobulbar (20 μL 10 mM TPCA-1 or control vehicle) or intravitreal (2 μL 10 mM TPCA-1 or control vehicle) injection immediately after laser injury and, seven days later, the FITC-dextran stained images were taken and calculated. (D) The size of CNV area developed in the laser-induced and TPCA-1 treated eyes was normalized to the size of CNV area in the laser-induced, and control solvent vehicle injected eyes. The TPCA-1 treatment was given once to the mice via either retrobulbar (20 μL 10 mM TPCA-1 or control vehicle) or intravitreal (2 μL 10 mM TPCA-1 or control vehicle) injection at 3 days after laser injury. The images were taken and calculated at 7 days after laser injury. (E) Comparison of the relative sizes of CNV area in the laser-injured mice that had been treated with either retrobulbar injection of 20 μL of 10 mM TPCA-1 or intravitreal injection of 2 μL of 2 μg/μL mouse VEGF 164 affinity purified polyclonal antibody. They were normalized to the size of CNV area in the laser-injured mice that had been treated with control solvent vehicle and IgG isotype control antibody, respectively. Error bars indicate standard deviation. Two-tail T-test: * *p*<0.05; ** *p*<0.01, *** *p*<0.005. “n” indicates the number of mice used at each condition.

To explore the therapeutic potential of IKK2 inhibitors in treating CNV, we next tested the effects of a potent (IC_50_ = 17.9 nM) and selective IKK-2 inhibitor, 2-[(aminocarbonyl)amino]-5-(4-fluorophenyl)-3-thiophenecarboxamide (TPCA-1) _ENREF_49[28], on laser-induced CNV. One eye of each mouse was treated with laser burns, followed by a single retrobulbar or intravitreal injection of either TPCA-1 or the control vehicle. As shown in figure 5A, a single retrobulbar injection of 20 μL TPCA-1 at 1, 5, 10, or 20 mM, resulted in a dose-dependent reduction in laser-induced CNV, with 5 mM being the minimum concentration to show an inhibitory effect (Fig. 5A). The average area of CNV spots in the TPCA-1 treated eyes was significantly reduced, especially in both the 10 mM (56 μg) and 20 mM (112 μg) groups, being approximately one third of the CNV area in control eyes (Fig. 5A). Clinical examination of the retina and OKR (on days 6 and 21) post retrobulbar injection of 20 μl TPCA-1 was normal (Fig. 5B, Figure S2 in File S1). Importantly, mice injected with control solvent either retrobulbarly or intravitreally showed no significant difference in laser-induced CNV in WT mice, indicating that the surgical procedure itself did not affect the incidence of laser-induced CNV (Fig. 5C, vehicle vs. WT). We observed that retrobulbar injection achieved a similar inhibitory effect as intravitreal injection, although the retrobulbar route required a larger injection volume (20 μl of 10 mM TPCA-1 compared to 2 μl of 10 mM TPCA-1) (Fig. 5C). We also observed that the inhibitory effect of retrobulbar injected 10 mM TPCA-1 was comparable to intravitreal anti-mouse VEGF 164 polyclonal antibody (R&D System, Cat#AF-493-NA) with regard to CNV size (Fig. 5E).

We noticed that the laser burn causes a continuous influx of inflammatory cells into the injury site with the peak at 3 days post-laser, the same time interval required for the onset of neovascularization [29]. To test if TPCA-1 will inhibit CNV development after neovascularization has started, we gave TPCA-1 3 days after the laser burn. A similar inhibitory effect on CNV was observed with either retrobulbar or intravitreal injection (Fig. 5D). These data show that the IKK2 inhibitor, TPCA-1, can inhibit CNV whether administered prior to or after the onset of angiogenesis, suggesting that IKK2 is critical for the expression of trophic factor(s) important for aberrant neovascularization.

### Local administration of TPCA-1 shows no systemic toxicity

**Figure 6:**
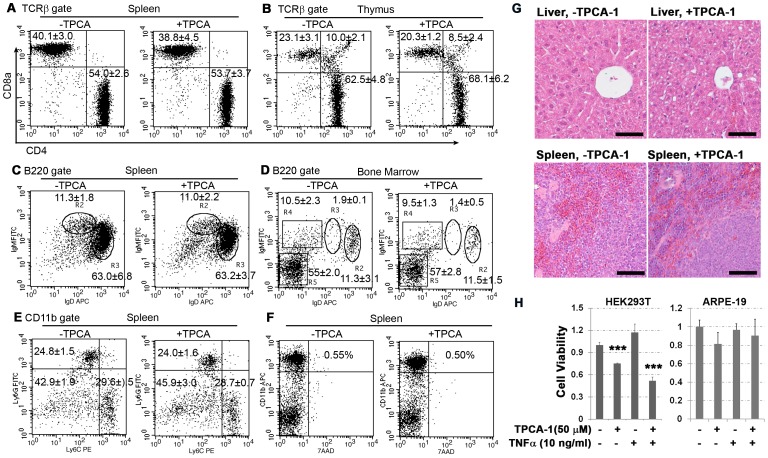
Retrobulbar administration of TPCA-1 causes no obvious toxicity to blood cells. (A–F) Flow cytometric analyses of blood cell population. Single cell suspension was prepared from spleen, thymus and bone marrow of the mice at 7 days after laser-injury followed immediately by a single retrobulbar injection of 56 μg TPCA-1 in 20 μL vehicle solution (PBS/20% DMSO) or control vehicle only in 20 μL. The representative plots are shown and numerical values represent the average (±SD, n>5). Statistics were performed using ProStat 5.5 program, and showing no significant difference in all the groups. P≤0.05 is considered as significant. (A, B) The CD4^+^ and CD8a^+^ T cells from the TCRβ-gated populations in spleen (A) and thymus (B) were calculated and plotted. (C, D) Spleen (C) and bone marrow (D) cells were isolated from TPCA-1 treated (+) and untreated (−) mice and stained with anti-B220, anti-IgM, and anti-IgD antibodies. Data in (C) show the percentile of mature (IgD^high^IgM^low^) and immature (IgD^low^IgM^high^) B cells in the B220-gated splenocytes, and data in (D) show the percentages of mature recirculating (R2, IgD^high^IgM^int^), transitional immature (R3, IgM^high^IgD^int^), immature (R4, IgM^high^IgD^−^) and precursor (R5, IgM^−^IgD^−^) B cell populations in the B220-gated bone marrow cells. (E) Spleen cells were isolated from TPCA-1 treated (+) and untreated (−) mice and stained with anti-CD11b, anti-Ly6C and anti-Ly6G antibodies. The numerical values are shown as the average of neutrophil (Ly6G^+^/Ly6C^int^), monocyte (Ly6G^−^/Ly6C^+^) and mature macrophage (Ly6G^−^/Ly6C^−^) from the CD11b gate in the spleens. (F) Spleen cells were stained with 7AAD to detect dead cells. The numerical values are shown as the average of the CD11b^+^ 7AAD^+^ populations in splenic lymphoid cells. (G) Hematoxylin and eosin (H&E) histological staining was performed to evaluate toxicity to liver and spleen at 7 days after treatment. The scale bar represents 50 μm in length. (H) Relative cell viability of HEK293T cells (left panel) and ARPE-19 cells (right panel) was shown at 12 hours after being cultured with 50 μM TPCA-1 or 10 ng/mL TNFα or combination of both. The cells were pretreated with 50 μM TPCA-1 for 30 minutes prior to addition of 10 ng/mL TNFα for combination treatment. Cell viability was measured using MTT assay. Data are represented as the mean±SD from three independent experiments. Asterisk indicates Two-tail T-test: *** *p*<0.005.

It has been previously shown that systemic administration of the IKK2 inhibitor ML120B for four days caused depletion of bone marrow B cells and thymocytes due to increased TNF-α-mediated apoptosis of lymphocytes [30]. Similar toxicity was observed in mice in which lethally irradiated hosts were transplanted with either IKK2- or p65-deficient fetal liver cells [31], [32]. In order to assess the potential toxicity of retrobulbar TPCA-1 we used flow cytometry to analyze systemic lymphoid tissues. A single retrobulbar injection of 56 μg TPCA-1 for 7 days did not alter the proportion of CD4^+^/CD8^+^ double and single positive T cells in both spleen and thymus (Fig. 6A–B). TPCA-1 treated mice showed no obvious alteration in mature (IgD^high^IgM^low^) or immature (IgD^low^IgM^high^) B220^+^ B-cell populations in the spleen (Fig. 6C), or in mature recirculating (B220^+^IgD^high^IgM^int^), transitional immature (B220^+^IgM^high^IgD^int^), immature (B220^+^IgM^high^IgD^−^) or precursor (B220^+^IgM^−^IgD^−^) B cells in the bone marrow either (Fig. 6D). There was also no significant difference noticed in the monocyte populations in the spleen of TPCA-1 treated mice - i.e. neutrophil (CD11b^+^Ly6G^+^/Ly6C^int^), monocyte (CD11b^+^Ly6G^−^/Ly6C^+^) and mature macrophage (CD11b^+^Ly6G^−^/Ly6C^−^) cells (Fig. 6E). Staining of CD11b and CD4 positive cells with 7AAD showed no increase in cell death (Fig. 6F). Thus, our data suggested that periocular (i.e. retrobulbar) administration of the IKK2 inhibitor, TPCA-1, did not cause systemic toxicity to the immune system. In addition, histological examination showed no noticeable morphological changes or tissue toxicity in the liver and spleen of the TPCA-1 treated mice (Fig. 6G).

**Figure 7:**
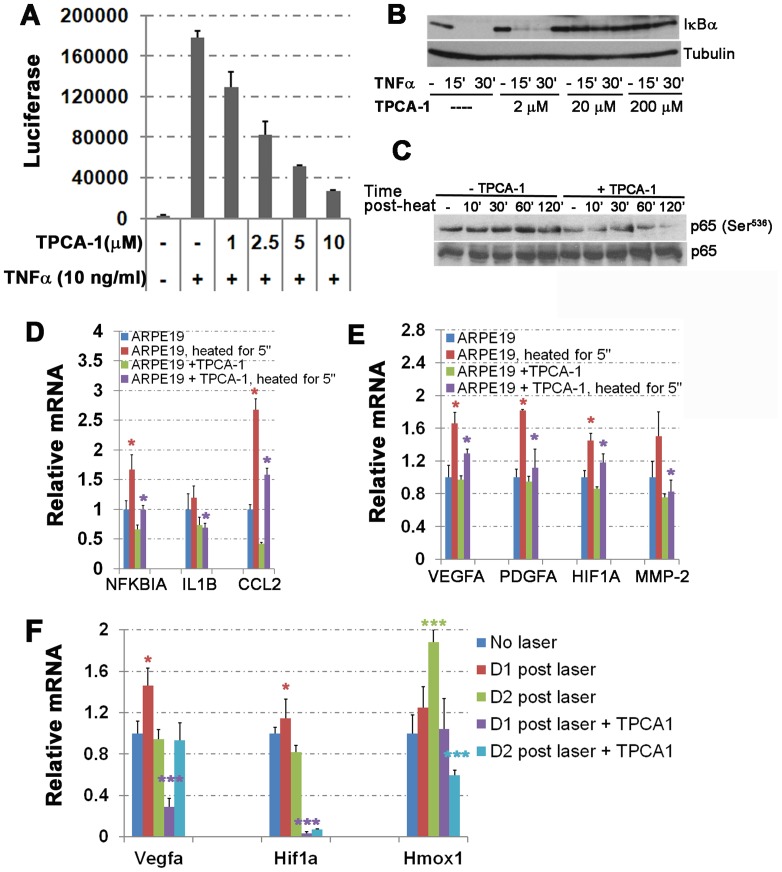
TPCA-1 inhibits NF-κB signaling pathway and the thermal injury-induced angiogenesis-associated factors. (A) TPCA-1 inhibits the TNFα-induced κB-Luciferase activity in a dosage dependent manner. 293T cells containing κB-Luciferase reporter were pretreated with or without TPCA1 for 30 minutes, then stimulated with 10 ng/mL TNFα to induce κB-Luciferase expression in the presence of the indicated amount of TPCA-1 for 5 hours, and followed by cell lysis and luciferase assay. (B) TPCA-1 inhibits TNFα-induced IκBα degradation in a dosage dependent manner. ARPE-19 cells were pretreated with TPCA-1 at indicated concentration for 30 minutes prior to addition of 10 ng/mL of TNFα for 15 and 30 minutes in the presence of the same amount of TPCA-1. Protein lysates were analyzed for the levels of IκBα and tubulin (as a loading control) by Western blotting. (C) TPCA-1 treatment inhibits the thermal injury-induced p65 phosphorylation at serine 536 amino acid residue. The ARPE-19 cells at 80% confluence in the complete DMEM/F-20 medium were pretreated with 5 μM TPCA-1 for 30 minutes and followed by heat shock for 5 seconds with addition of 10 mL HEPES-buffered saline preheated at 55°C; and then the heating medium was immediately replaced with normal complete DMEM/F-20 medium containing 5 μM TPCA-1 and further cultured at 37°C for 10, 30, 60, and 120 minutes before the protein lysates were collected. Western blotting was used to detect the total NF-κB p65 protein and the activated form of NF-κB p65 phosphorylated at Serine 536. (D and E) TPCA-1 treatment reduces thermal injury-induced and NF-κB dependent transcription of signaling protein, cytokine and chemokine, such as IκBα, IL1B and CCL2 (D), as well as of those associated with angiogenesis, such as VEGF-A, PDGFA, HIF-1A, and MMP-2 (E) in ARPE-19 cells. Cells were treated similarly as described in C. Four hours after the thermal treatment, RNAs were isolated for qPCR analysis. Relative mRNA levels were normalized to β-actin first, and then the fold increase or decrease by heat shock. Cells were treated with heat shock at the presence or absence of TPCA-1 or treated with TPCA-1 without heat shock were compared to control cells without heat shock and TPCA treatment. Error bars indicate standard deviation. Two-tail T-test: * *p*<0.05, n = 3. The red * indicates the comparison between the heat shocked cells and control cells. The purple * indicates the comparison between the heat shocked cells at the presence or absence of TPCA-1. (F) Relative mRNA level of Vegfa, Hif1a, and Hmox1 was shown in the RPE/Choroid/Sclera tissues collected from laser injured eyes treated with or without TPCA-1. Mice received the retrobulbar injection of 56 μg TPCA-1 or vehicle solution and the RNA samples were prepared from RPE/Choroid/Sclera tissues at day 1 and day 2 post injury. Each RNA sample was pooled from 4 eyes in the same condition and the control sample is from the eyes without laser injury. Red and green * show the significant difference between the indicated sample and control sample without laser injury. Purple and light blue *** show the significant difference between the laser injured samples at the presence or absence of TPCA-1. Error bars indicate standard deviation. Two-tail T-test: * *p*<0.05, *** *p*<0.005, n = 3.

It is well documented that NF-κB inhibition enhances TNFα-induced apoptosis in HEK293T cells. Similarly, TPCA-1 significantly reduced the viability of HEK293 cells, and the addition of TNFα increased cell death (Fig. 6H left panel), suggesting TPCA-1 sensitized HEK293T cells to TNFα induced apoptosis. We then asked whether TPCA-1 was also able to affect ARPE-19 cell viability. In contrast to HEK293T cell, TPCA-1 showed no toxicity for ARPE-19 cells (Fig. 6H, right panel), even at the higher concentration of 50 μM which specifically blocked NF-κB activation in both HEK293T and ARPE-19 cells (Fig. 7A–B).

### TPCA-1 attenuates thermal injury-induced expression of angiogenic factors

Given that activation of IKK-NF-κB signaling induced expression of growth factors and chemokines associated with angiogenesis [33], we investigated whether TPCA-1 inhibited laser-induced CNV through down-regulation of other inflammatory and angiogenic factors that promoted neovascularization. It is well known that TNF-α activates the IKK-NF-κB signal transduction pathway, leading to transcriptional activation of downstream target genes. To determine an optimal inhibitory dose for TPCA-1 to block NF-κB activation, we first performed a dose-dependent inhibition assay in HEK293T cells using a commercially available κB luciferase assay system (Promega). The κB-luciferase activity induced by TNFα was significantly inhibited by TPCA-1 at 1 μM, and was dose dependent and reaching a peak at the highest dosage used in this assay (10 μM, approximately a 7-fold reduction) (Fig. 7A). We next examined the inhibitory effect of TPCA-1 on ARPE-19 cells by visualizing TNFα-induced IκBα degradation on Western blots. In general, TNFα activates IKK2 which phosphorylates its downstream IκBα serine residues at 32 and 36, leading to ubiquitin-dependent degradation of this inhibitory protein. Degradation of IκBα releases the NF-κB dimer into the nucleus to activate downstream transcriptional targets [34]. When ARPE-19 cells were treated with 10 ng/mL TNFα, endogenous IκBα protein was rapidly degraded within 15 minutes (Fig. 7B). In contrast, pre-incubation of the cells with 20 μM and 200 μM TPCA-1 completely blocked TNFα-induced IκBα degradation (Fig. 7B).

We next explored the inhibitory effect of TPCA-1 on NF-κB activation induced by heat shock to mimic the laser injury [35]. Human ARPE-19 cells were pretreated with or without 5 μM TPCA-1 for 30 minutes and were quickly shocked by heat at 55°C for 5 seconds; they were then cultured in the presence or absence of 5 μM TPCA-1 for various times. The phosphorylation of NF-κB p65 subunit at Ser536, which mediates IκB release and DNA binding, was analyzed by Western blotting. Heat shock induced a slight increase in p65 phosphorylation at Ser 536, starting at 30 minutes after heating; however, such heat shock-induced p65 phosphorylation was significantly reduced in the presence of TPCA-1, suggesting that TPCA-1 blocked the heat shock-induced NF-κB activation (Fig. 7C). Furthermore, to examine whether TPCA-1 affected heat shock-induced gene expression, we heat-shocked ARPE-19 cells at 55°C for 5 seconds. Total RNA was isolated from those cells at 4 hrs and subjected to qPCR analysis of downstream target genes. As shown in figure 7D and 7E, heat shock significantly induced expression of NFKBIA, IL1B, CCL2, VEGF-A, PDGFA, HIF1A, and MMP-2, all of which have been shown to be regulated by NF-κB signaling and participate in regulation of angiogenesis. Notably, TPCA-1 significantly blocked the activation of these downstream genes (Fig. 7D and 7E). Next, we confirmed that mRNA expression of Vegfa and Hif1a was induced by the laser injury and that their expression was suppressed by TPCA-1 *in vivo* (Fig. 7F). In addition, the induced heme oxygenase enzyme HO-1 isoform (Hmox1), a sensor of cellular oxidative stress, was also significantly induced by laser injury and its response was also blocked by TPCA-1, suggesting that NF-κB/IKK2 regulates laser induced CNV formation through multiple pathways including inflammation, hypoxia, and oxidative stress (Fig. 7F). Our results suggest that IKK2 inhibition by its specific inhibitor TPCA-1 is able to block laser-induced CNV through the IKK-NF-κB signaling pathway, and in turn, the transcription of its downstream target genes that promote neovascularization.

## Discussion

Abnormal growth of choroidal blood vessels into the subretinal space (i.e. CNV) is a major cause of severe vision loss in AMD. The most effective current treatment for CNV is intravitreal injection of antibodies that block the action of VEGF [2]. Because over 60% of AMD patients with CNV do not have improved vision after anti-VEGF antibody treatment, other alternative treatments are being sought. Inflammation has been linked to the development of CNV and NF-κB is a master regulator of inflammation. Therefore, we explored the effect of inhibiting the NF-κB signaling pathway in an experimental model of CNV induced by laser injury to the retina. We observed in the present study that inhibition of IKK2/NF-κB activation by the pharmaceutical IKK2 inhibitor TPCA-1 or using *Ikk2* conditional knockout mice dramatically inhibited laser-induced CNV.

Clinical evidence shows that in many patients VEGF is critical for endothelial cell proliferation and angiogenesis that leads to CNV. VEGF consists of several isomers (VEGF-A, VEGF-B, VEGF-C, and VEGF-D) that have a different ability to promote angiogenesis [36], [37]. Increased levels of the VEGF protein has been detected in RPE cells of CNV membranes in AMD [7], [38]–[41]; in basal laminar deposits of surgically excised CNV membranes; in vitreous samples of patients with neovascular retinopathies [7], [39], [40]. In addition, VEGF is known as a vascular permeability factor that can increase blood vessel leakage by interrupting endothelial layer tight junctions. Multiple anti-VEGF drugs are beneficial in inhibiting CNV and restoring visual function. The production of VEGF and PDGF, and the activation of multiple pro-inflammatory gene products (e.g. CCL2, ICAM-1, IL1B and IL-8) are regulated by the transcription nuclear factor NF-κB [42]–[46]. Thus, inhibition of the NF-κB pathway by pharmaceutical intervention may open a novel approach to prevent CNV [22].

Mounting evidence indicates that NF-κB also functions as a regulator of angiogenesis promoting tumor growth by upregulating the expression of angiogenic factors, such as IL-8, CCL2 and VEGF [47], [48]. It has been documented that NF-κB is involved in the upregulation of VEGF mRNA in multiple cancer cell lines [49]. Although the VEGF promoter contains a region with putative NF-κB binding sites, deletion of these binding sites had little effect on VEGF expression, suggesting that direct binding of NF-κB to the VEGF promoter may not be required for its expression [50]. However, it was recently proposed that NF-κB modulates VEGF transcription though HIF1A, a key transcription factor for VEGF during hypoxia [16], [51], and a recent study showed that transcriptional activation of the Hif1a gene by IKK2-activated NF-κB precedes HIF1A protein accumulation both *ex vivo* and *in vivo* [52]. Mice lacking *Ikk2* subjected to hypoxia revealed a pronounced defect in Hif1a mRNA expression and protein accumulation, as well as defective induction of HIF1A targeted genes, including VEGF [52].

We hypothesize that injury or inflammation in the laser injured retina may induce aberrant angiogenesis through the release of angiogenic factors that are regulated directly by NF-κB similar to that demonstrated for tumor cells [52]–[59]. We observed that TPCA-1, a selective inhibitor of IKK2, led to significant suppression of several pro-angiogenic molecules including IL1B, CCL2, VEGFA, PDGFA, HIF1A, and MMP-2 (Fig. 7). Furthermore, we also observed the inhibition of laser-induced CNV in genetic mouse mutants in which *Ikk2* was selectively deleted in RPE cells and retinal neurons but not in the vascular endothelium.

Inhibition of the NF-κB activation pathway has gained increasing interest in the treatment of several diseases including asthma, chronic obstructive pulmonary disease, and inflammation-associated cancer [60], [61] because of its anti-inflammatory effects. IKK2, the proximal kinase leading to NF-κB activation, is an excellent target for inhibition of this pathway [13]. However, oral and systemic delivery of these inhibitors has been shown to be toxic to bone marrow and epithelial cells [30], [62]–[64]. Therefore, local delivery of this drug to the eye is an attractive therapeutic alternative. Our data showed that retrobulbar injection of 56 μg TPCA-1 inhibited laser-induced CNV in mice and had no detrimental effect to liver, spleen and bone marrow derived cells (Fig. 6A–G), as well as skin epithelium (data not shown). Consistent with a previous report [65], IKK2 inhibition by TPCA-1 did not cause ARPE-19 cell death even with TNFα induction (Fig. 6H). In addition, neither genetic deletion of *Ikk2* gene nor periocular administration of TPCA-1 showed an adverse effect on retinal histology or visual function (Fig. 3 and Fig. 5B).

Anti-VEGF therapy for CNV results in significant vision gain in approximately one-third of patients with wet AMD mostly due to its anti-permeability effect but not the inhibition of aberrant angiogenesis [66]–[68]. In search of better treatments, a multi-targeted therapeutic approach has been proposed and a recent clinical study using anti-PDGF and anti-VEGF combination therapy in wet AMD has reported improved visual outcome compared with anti-VEGF monotherapy [69_ENREF_69]. Targeting multiple signaling pathways involved in CNV may be a better option to improve therapeutic results. As a key regulator of several factors promoting inflammation and angiogenesis, IKK2/NF-κB inhibition is a rational therapeutic strategy. Another advantage of IKK2/NF-κB inhibition therapy over anti-VEGF monotherapy is the targeting of an earlier phase of angiogenesis by inhibiting inflammation. In summary, we have demonstrated that periocular injection of TPCA-1, a selective IKK2 inhibitor, is able to reduce the development of laser-induced CNV by blocking activation of the NF-κB signal pathway that is important for expression of multiple inflammatory and angiogenic factors. Our results suggest that IKK2 inhibition could be a potential novel approach to prevent or reduce CNV formation.

## Supporting Information

File S1 Contains: Figure S1 Fluorescein angiography of laser induced CNV and size grading for fluorescein leakage. (A) Lesion sizes were graded based on the relative comparison to the optic nerve disk area. (B–C) Lesion sizes in the same eye were graded at 3 and 6 minutes after intraperitoneal injection of fluorescein. Figure S2 Fundus photography before and after retrobulbar injection. (A–A1) the fundus images of a mouse eye before and after retrobulbar injection of 50 μL of PBS/20% DMSO. (B–B1) the fundus images of another eye before and after retrobulbar injection of 50 μL of PBS/20% DMSO.
(DOCX)
