# Supplementary material for: IKK2 Inhibition Attenuates Laser-Induced Choroidal Neovascularization
Source: PLoS One. 2014 Jan 28;9(1):e87530. doi: 10.1371/journal.pone.0087530 (PMC3905033; doi:10.1371/journal.pone.0087530)
Supplement: File S1 — Contains: Figure S1 Fluorescein angiography of laser induced CNV and size grading for fluorescein leakage. (A) Lesion sizes were graded based on the relative comparison to the optic nerve disk area. (B–C) Lesion sizes in the same eye were graded at 3 and 6 minutes after intraperitoneal injection of fluorescein. Figure S2 Fundus photography before and after retrobulbar injection. (A–A1) the fundus images of a mouse eye before and after retrobulbar injection of 50 μL of PBS/20% DMSO. (B–B1) the fundus images of another eye before and after retrobulbar injection of 50 μL of PBS/20% DMSO. (DOCX) [file pone.0087530.s001.docx]

**Supporting information**

**Figure S1** **Fluorescein angiography of laser induced CNV and size grading for fluorescein leakage.** (A) Lesion sizes were graded based on the relative comparison to the optic nerve disk area. (B-C) Lesion sizes in the same eye were graded at 3 and 6 minutes after intraperitoneal injection of fluorescein.

**Figure S2** **Fundus photography before and after retrobulbar injection.** (A-A1) the fundus images of a mouse eye before and after retrobulbar injection of 50 µL of PBS/20% DMSO. (B-B1) the fundus images of another eye before and after retrobulbar injection of 50 µL of PBS/20% DMSO.

Figure S1





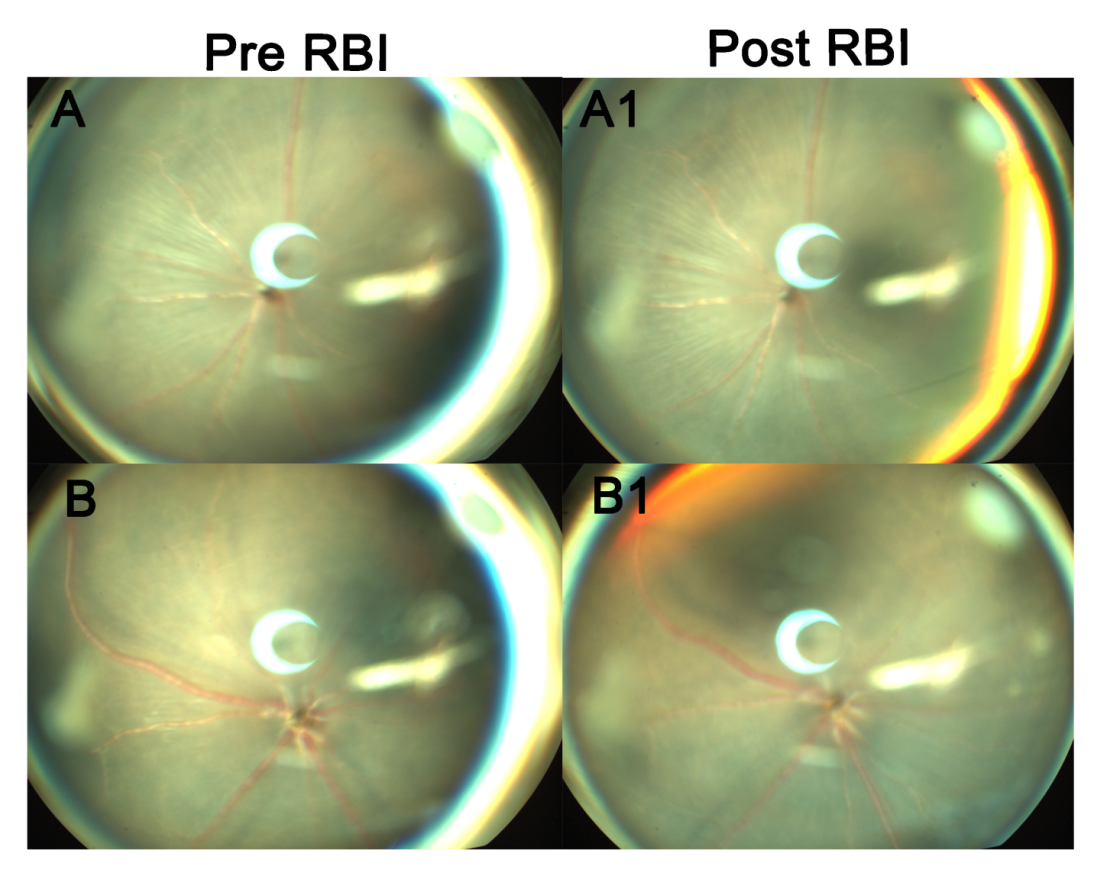
Figure S2
